# Below- versus above-elbow cast treatment of displaced distal forearm fractures in children: A systematic review and meta-analysis of randomized controlled trials

**DOI:** 10.1177/18632521231162621

**Published:** 2023-06-01

**Authors:** Osama Z Alzobi, Ashraf T Hantouly, Mohamed Kenawey, Talal Ibrahim

**Affiliations:** 1 Department of Orthopaedic Surgery, Hamad Medical Corporation, Doha, Qatar; 2 Orthopaedic Department, Royal Manchester Children’s Hospital, Manchester University NHS Foundation Trust, Manchester, UK; 3 Orthopaedic Department, Faculty of Medicine, Sohag University, Sohag, Egypt; 4 Division of Orthopaedic Surgery, Department of Surgery, Sidra Medicine, Doha, Qatar

**Keywords:** Displaced distal forearm fractures, pediatrics, cast, randomized controlled trial, meta-analysis

## Abstract

**Objectives:**

Distal forearm fractures are the most common pediatric fractures. This study aimed to investigate the effectiveness of below-elbow cast treatment for displaced distal forearm fractures in children compared to above-elbow cast through meta-analysis of randomized controlled trials.

**Methods:**

Several databases from January 1, 2000 until October 1, 2021 were searched for randomized controlled trials that assessed below versus above-elbow cast treatment of displaced distal forearm fractures in pediatric patients. The main meta-analysis comparison was based on the relative risk of loss of fracture reduction between children undergoing below versus above-elbow cast treatment. Other outcome measures including re-manipulation and cast-related complications were also investigated.

**Results:**

Nine studies were eligible of the 156 articles identified, with a total of 1049 children. Analysis was undertaken for all included studies with a sensitivity analysis conducted for studies with high quality. In the sensitivity analysis, the relative risks of loss of fracture reduction (relative risk = 0.6, 95% confidence interval = 0.38, 0.96) and re-manipulation (relative risk = 0.3, 95% confidence interval = 0.19, 0.48) between the below and above-elbow cast groups were in favor of below-elbow cast and statistically significant. Cast-related complications were in favor of below-elbow cast but did not attain statistical significance (relative risk = 0.45, 95% confidence interval = 0.05, 3.99). Loss of fracture reduction was noted in 28.9% of patients treated with above-elbow cast and 21.5% in below-elbow cast. Re-manipulation was attempted in 48.1% versus 53.8% of children who lost fracture reduction in the below-elbow cast and above-elbow cast groups, respectively.

**Conclusion:**

Below-elbow cast treatment was favored, with statistical significance, in terms of loss of fracture reduction and re-manipulation, and was not associated with a higher risk of cast-related complications. The accumulative evidence currently does not support above-elbow cast treatment and below-elbow cast treatment should be the mainstay for displaced distal forearm fractures in children.

**Level of evidence:**

Level I, meta-analysis of therapeutic level I studies.

## Introduction

Distal third forearm metaphyseal fractures are the most common injuries in children. These fractures represent 40% of all fractures in children and adolescents.^1,2^ Despite this high prevalence, there is no unequivocal evidence regarding the optimal immobilization method when such fractures are treated non-surgically.^3,4^

Displaced distal forearm fractures are generally reduced in emergency settings under conscious sedation and stabilized in a cast. Immobilization of such fractures in children is usually managed with either above-elbow cast (AEC) or below-elbow cast (BEC).^5^ AEC controls both elbow and forearm range of motion by neutralizing muscles forces that originate above the elbow and is thought to reduce the risk of displacement and loss of reduction of such fractures.^6,7^ Whereas, BEC has been proposed as a valid alternative that is easier to apply, allows better hand function, and minimize elbow stiffness.^8,9^ However, in children with either BEC or AEC treatment, the initial reduction is not always successful, and loss of fracture reduction (25%–39%) and re-manipulation during the first few weeks are frequent complications.^10,11^

Several published studies have compared different outcome measures for BEC and AEC treatment. While this is not the first meta-analysis to report on BEC versus AEC treatment for displaced distal forearm fractures in pediatric patients, we decided to perform this meta-analysis because of the inconsistent results regarding the direction and scale of differences in the studies that reflect the existing uncertainties. Hendrickx et al.^12^ published a meta-analysis more than 10 years ago that included three randomized controlled trials (RCTs). An update of the meta-analysis^13^ was further published in 2012, which included an additional two RCTs.^14,15^ Due to heterogeneity, the meta-analysis failed to generate robust conclusions and hence has provoked additional RCTs to answer this question. Since the updated meta-analysis, four additional recent RCTs^16–19^ with larger sample sizes have been published.

Currently, there is no clear consensus regarding the treatment of displaced distal forearm fractures in children. This meta-analysis aimed to evaluate the effectiveness of BEC treatment for displaced distal forearm fractures in pediatric patients compared to AEC. Analysis of outcomes included loss of fracture reduction, re-manipulation, and cast-related complications. We hypothesized that BEC treatment would have similar rates of loss of fracture reduction, re-manipulation, and cast-related complications compared to AEC.

## Materials and methods

This meta-analysis was conducted with strict adherence to the guidelines of the Preferred Reporting Items for Systematic Reviews and Meta-Analyses (PRISMA).^20,21^

## Search method and strategy

PubMed/MEDLINE, Web of Science, Cochrane Library, and Google Scholar were searched from January 1, 2000 until October 1, 2021. We used the following keywords: (“distal radius” or “forearm” or “wrist”) and (“long” or “below”) and (“Short” or “below”) and (“Pediatrics” or “children”) and “Randomized.” Articles were confined to RCTs of children. Two authors independently screened the titles and abstracts, and a full-text review was conducted for eligible studies.

## Eligibility criteria

Inclusion criteria were RCTs that compared BEC to AEC treatment of displaced distal forearm fractures (including displaced distal radius fractures with or without distal ulna fractures) in the pediatric population (age < 16 years) and reported the rates of loss of fracture reduction, re-manipulation, and cast-related complications. The exclusion criteria entailed non-comparative studies, published studies in languages other than English, studies that included surgical management of distal forearm fractures in pediatric patients, and studies that included dislocation of the distal radial-ulna joint, radial head fractures, or isolated distal ulna fractures.

## Data extraction methods

Two authors independently collected data from the selected studies using a spreadsheet with a standardized approach. The collected items were as follows: study characteristics (i.e. country of origin, study year), number of patients undergoing BEC versus AEC treatment, sample size, age of children, bone involvement, follow-up duration, loss of follow-up, displacement criteria, loss of fracture reduction rate, re-manipulation rate, and cast-related complications.

## Quality assessment and risk of bias

The revised Cochrane risk-of-bias tool for randomized trials (RoB 2) was utilized to conduct the qualitative assessment.^22,23^ This tool comprises five domains as follows: randomization, adherence to treatment, missing outcomes, bias measurement, and bias reporting. Two authors assessed the methodological quality of the included studies with this tool independently, and where disagreement existed, a consensus was reached.

## Statistical analysis

Using a random-effects model, studies and constructed Forest plots were pooled. The random-effects model identifies articles as a sample of all potential articles and incorporates a between-article random-effect element to allow for between-study heterogeneity. We attained the rates of loss of fracture reduction, re-manipulation, and cast-related complications for each study. Between-study heterogeneity was quantified using the *I*^2^ statistic. This elucidates the variability percentage in effect estimates that is owed to heterogeneity instead of chance.

The main meta-analytic comparison was based on the rate of loss of fracture reduction in children who were treated with BEC versus those in AEC. The rates of re-manipulation and cast-related complications were also compared. Overall relative risks (RRs) were calculated. The RRs (risk ratios) were calculated as the effect size metric and pooled using the Mantel–Haenszel method without continuity correction. τ2 was estimated using the Paule–Mandel estimator. τ and τ2 confidence intervals were estimated using the Q-profile method. The Hartung–Knapp adjustment was utilized for random-effects model. Continuity correction of 0.5 in studies with zero cell frequencies (only utilized to measure individual studies results). Sensitivity analysis was conducted with the exclusion of studies associated with high risk of bias.^15,17,19^

Another sensitivity analysis was conducted to evaluate if excluding articles^9,14,16^ with children elder than 12 years of age would affect the results on the basis that these studies had adolescent patients compared with other studies with children younger than 12 years of age. Egger’s test with funnel plot was performed for our primary outcome, the risk of loss of fracture reduction. RStudio (2021.09.0 + 351 “Ghost Orchid” Release) was used for all statistical analyses.

## Results

### Study selection

**Figure 1.**
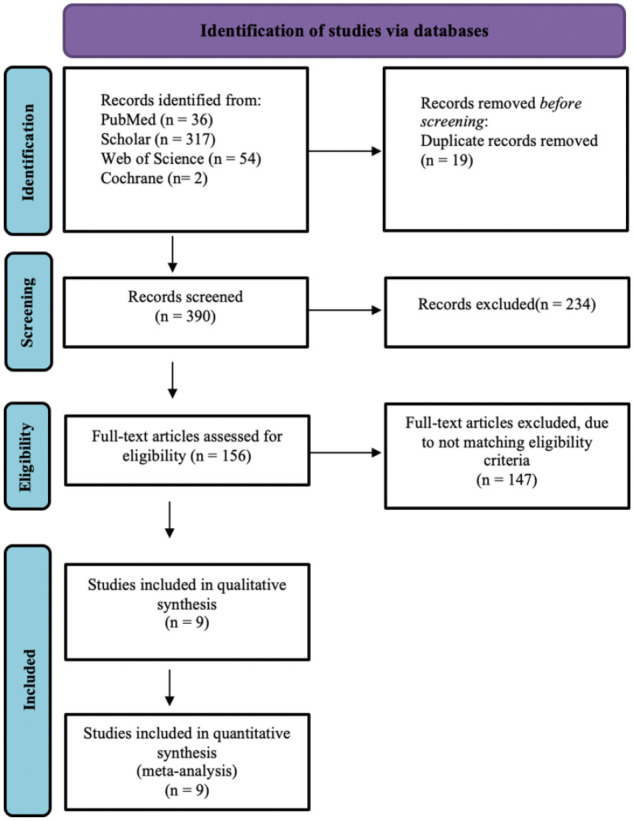
PRISMA flow diagram of the systematic search process detailing article identification, screening, eligibility, and inclusion steps along with reasons for exclusion.

The literature search yielded 390 publications. After screening using titles and abstracts, 156 articles were left for full-text review. After applying eligibility criteria, nine articles remained for the analysis addressing BEC versus AEC treatment of displaced distal forearm fractures in pediatric patients.^8,9,14–19,24^ One publication reported the long-term follow-up of the same study as the original RCT.^25^ The kappa statistics for interobserver agreement on study eligibility was 1.0. The detailed selection process (PRISMA diagram) is shown in Figure 1.

### Study and patient characteristics

Tables 1 and 2 summarize the characteristics of the nine included studies in our meta-analysis. The studies described a total of 1049 children (647 males and 402 females). Of those, 519 (49.5%) of whom were treated with BEC and 530 (50.5%) were treated with AEC.^8,9,14–19,24^ The mean age of all children was 8.4 years. Cast application time ranged from 4 to 6 weeks, whereas follow-up duration ranged from 6 weeks to 8 months. Some studies did not report all basic demographics, which limited grouped statistics. Eight out of the nine studies reported loss of fracture reduction rates. Memon et al.^18^ described only the mean loss of fracture reduction for each group, resulting in an incapability to pool data for the meta-analysis. Sensitivity analysis was conducted and excluded studies with high risk of bias (15, 17, 19). Seven of the studies reported re-manipulation rates and a total of five studies reported cast-related complications. Treatment protocols of the included studies in our meta-analysis are summarized in Table 3.

**Table 1.** Demographic characteristics of the included studies in the meta-analysis.

Author	Year of publication	Country	Mean age (years)		Number of patients		Outcomes	Follow-up
			BEC	AEC	BEC	AEC		
Bohm et al.^8^	2006	Canada	8.6	8.6	46	56	Loss of fracture reduction Re-manipulation Cast-related complications Residual angulation Cast index	18 weeks
Webb et al.^9^	2006	The United States	10.15	9.53	53	60	Fracture displacement Loss of fracture reduction Re-manipulation Cast index Wrist and elbow range of motion Questionnaire relating to the impact of the cast on activities in daily living	8 months
Paneru et al.^24^	2010	Nepal	8.05	8.76	43	42	Loss of fracture reduction Re-manipulation Cast-related complications Residual angulation Cast index Time to fracture union Lost school days Treatment cost	6 months
Rahman et al.^15^	2011	Pakistan	7.05	7.16	54	54	Loss of fracture reduction Re-manipulation Cast-related complications Time from injury to manipulation	6 weeks
Colaris et al.^14^	2012	The Netherlands	7.8	6.3	35	31	Limitation of elbow and wrist range of motion Loss of fracture reduction Re-manipulation Cast-related complications ABILHAND-kids questionnaire VAS comfort of cast VAS cosmetics by parents VAS cosmetics by surgeon	6 months
Hafeez et al.^19^	2018	Pakistan	8.05	8.05	114	111	Loss of fracture reduction Re-manipulation	Not stated
Manzoor et al.^17^	2019	Pakistan	7.38	7.66	90	90	Loss of fracture reduction	Not stated
Memon et al.^18^	2021	Pakistan	9.13	9.42	24	26	Loss of fracture reduction Cast-related complications	3 months
Seiler et al.^16^	2021	Switzerland	9.9	9.9	60	60	Loss of fracture reduction Re-manipulation Days of analgesic medication Assessment of restriction in activities of daily life Time to regain normal motion of the elbow	7 weeks

BEC: below-elbow cast; AEC: above-elbow cast; VAS: visual analogue scale; ABILHAND: a parent-reported questionnaire measuring manual ability for children with upper-limb impairments.

**Table 2.** Fracture characteristics of the included studies in the meta-analysis.

Author	Fracture type			
	Below-elbow cast		Above-elbow cast	
	Distal radius fracture only	Combined distal radius and ulna fractures	Distal radius fracture only	Combined distal radius and ulna fractures
Bohm et al.^8^	19	27	14	42
Webb et al.^9^	27	26	22	38
Paneru et al.^24^	0	43	0	42
Rahman et al.^15^	22	32	28	26
Colaris et al.^14^	0	35	0	31
Hafeez et al.^19^	74	40	71	40
Manzoor et al.^17^	−	−	−	−
Memon et al.^18^	16	8	11	15
Seiler et al.^16^	31	29	28	32

**Table 3.** Treatment protocol of the included studies in the meta-analysis.

Author	Fracture type	BEC	AEC	Duration of casting
Bohm et al.^8^	Displaced closed fracture of the distal third of the forearm (radius or combined radius and ulna fractures; no isolated distal ulna fractures)	Full below-elbow plaster cast	Above-elbow plaster cast (below-elbow applied first; then cast extended)	Casts removed after 6 weeks
Webb et al.^9^	Displaced (partially or completely) closed fracture of the distal third of the forearm (radius or combined radius and ulna fractures)	Full below-elbow plaster cast	Above-elbow plaster cast (below-elbow applied first; then cast extended)	Casts removed after 4 weeks if evidence of healing. Otherwise extended 2 weeks (above-elbow cast reduced to below-elbow cast)
Paneru et al.^24^	Displaced closed distal forearm fractures (combined radius and ulna fractures)	Full below-elbow plaster cast	Above-elbow plaster cast (below-elbow applied first; then cast extended)	Not stated
Rahman et al.^15^	Displaced distal third forearm fractures	Full below-elbow plaster cast	Above-elbow plaster cast (below-elbow applied first; then cast extended)	Casts removed after 6 weeks
Colaris et al.^14^	Minimally displaced metaphyseal fractures of the radius and ulna	Below-elbow plaster cast (non-circumferential)	Above-elbow plaster cast (non-circumferential)	Casts removed after 4 weeks
Hafeez et al.^19^	Displaced (partially or completely) closed fracture of the distal third of the forearm (radius or combined radius and ulna fractures)	Not stated	Not stated	Not stated
Manzoor et al.^17^	Displaced distal third forearm fractures	Not stated	Not stated	Not stated
Memon et al.^18^	Displaced (partially or completely) closed fracture of the distal third of the forearm (radius or combined radius and ulna fractures)	Full below-elbow plaster cast	Above-elbow plaster cast (below-elbow applied first; then cast extended)	Casts removed after 6 weeks
Seiler et al.^16^	Displaced distal third forearm fractures	Soft-Scotch cast with extension below the elbow combined with rigid fiberglass splint on the forearm	Soft–scotch cast with extension above the elbow combined with rigid fiberglass splint on whole arm dorsal and on the forearm volar	Casts removed after 4 weeks

BEC: below-elbow cast; AEC: above-elbow cast.

### Qualitative assessment

**Figure 2.**
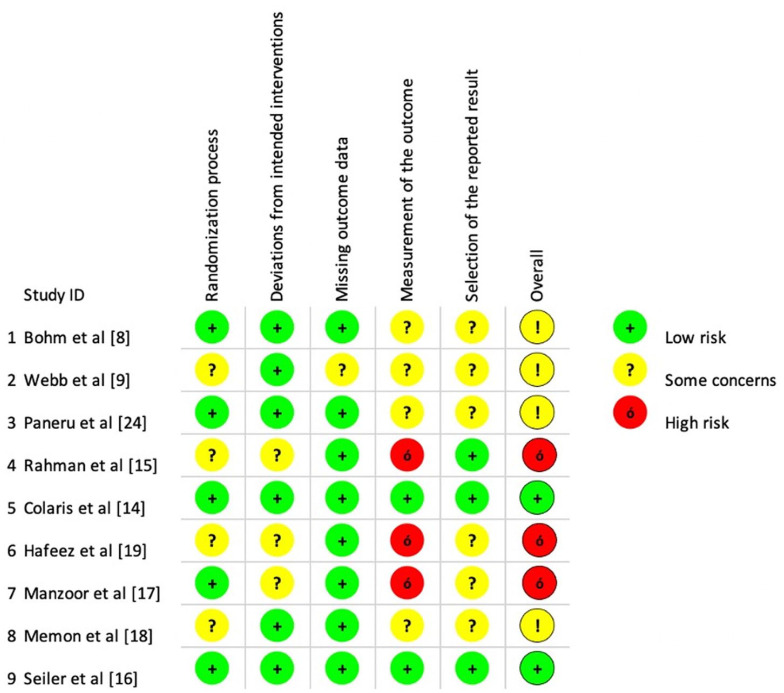
Summary charts for the risk of bias assessments for all included RCTs in all five different domains.

Figure 2 summarizes the risk of bias assessment for the included RCTs. Four studies were not clear about whether randomization was concealed before treatment. Patient blinding was not applicable in this study design. All studies evaluated groups according to original randomization. The loss to follow-up ranged from 2% to 8%. None of the included studies had a significant dropout rate or missed reporting outcomes. Overall bias was considered “Low” in two RCTs, “Some concerns” in four RCTs, and “High” in three RCTs.

### Loss of fracture reduction

**Figure 3.**
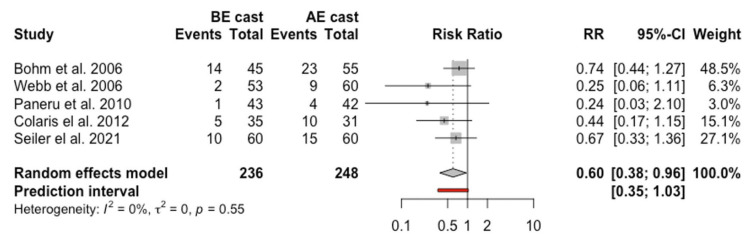
Forest plot: pooled relative risk for loss of fracture reduction for BEC versus AEC treatment.

Eight articles were incorporated in the analysis of the RR of loss of fracture reduction with a total of 994 children.^8,9,14–17,19,24^ A total of 106 out of 493 children in BEC group (21.5%) and 145 out of 501 children in AEC group (28.9%) had a loss of fracture reduction. The random-effects model meta-analysis of the eight RCTs concluded an overall RR of 0.74 (95% confidence interval (CI) = 0.49, 1.13, *p* = 0.07, *I*^2^ = 47%), which proposed a lower rate of loss of fracture reduction in children who had BEC treatment, but this difference did not reach a statistical significance (*p* = 0.14). However, sensitivity analysis following exclusion of studies with high risk of bias (15, 17, 19) showed that loss of fracture reduction was in favor of BEC with statistical significance (RR = 0.6, 95% CI = 0.38, 0.96) (Figure 3). Egger’s test did not indicate the presence of publication bias. Definitions of loss of fracture reduction are outlined in Table 4.

**Table 4.** Definitions of loss of fracture reduction.

Author	Isolated distal radius fractures	Combined distal radius and ulna fractures
Bohm et al.^8^	>25° of angulation on the lateral radiograph >10° of angulation on the posteroanterior radiograph <50% apposition on either the posteroanterior or lateral radiograph Shift of ≥15° in 1 week on the lateral radiograph	>10° of angulation of either bone on the lateral or posteroanterior radiograph <25% apposition of the fracture on the lateral or posteroanterior radiograph
Webb et al.^9^	>10° of angulation or deviation and >20% displacement compared with the postreduction values	>10° of angulation or deviation and >20% displacement compared with the postreduction values
Paneru et al.^24^	>10° of angulation and displacement >20%	>10° of angulation and displacement >20%
Rahman et al.^15^	>10° of angulation and displacement >20%	>10° of angulation and displacement >20%
Colaris et al.^14^	>15° of angulation in <10 years >10° of angulation in 10–16 years Increase translation half of bone diameter in <16 years Increase rotation >0° in <16 years	>15° of angulation in <10 years >10° of angulation in 10–16 years Increase translation half of bone diameter in <16 years Increase rotation >0° in <16 years
Hafeez et al.^19^	–	–
Manzoor et al.^17^	–	–
Memon et al.^18^	>25° of angulation on the lateral radiograph >10° of angulation on the posteroanterior radiograph <50% apposition on either the posteroanterior or lateral radiograph Shift of ≥15° in 1 week on the lateral radiograph	>10° of angulation of either bone on the lateral or posteroanterior radiograph <25% apposition of the fracture on the lateral or posteroanterior radiograph
Seiler et al.^16^	>20° of angulation in the lateral view in <11 years >10° of angulation in the lateral view in 12–16 years	>20° of angulation in the lateral view in <11 years >10° of angulation in the lateral view in 12–16 years

### Re-manipulation

**Figure 4.**
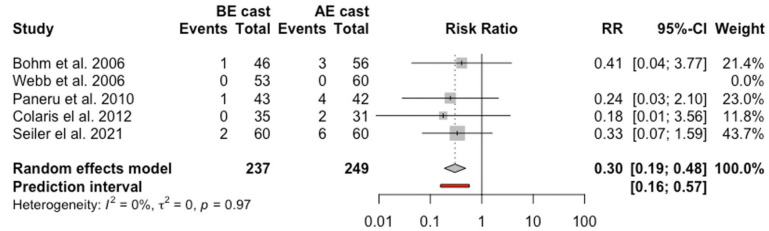
Forest plot: pooled relative risk for re-manipulation for BEC versus AEC treatment.

Seven studies with 816 children were included in the analysis of the re-manipulation with 404 in the BEC group and 412 in the AEC group.^8,9,14–16,19,24^ Fifty-one children (12.6%) from the BEC group and 78 patients (18.9%) from the AEC underwent re-manipulation. The overall RR was 0.66 (95% CI = 0.36, 1.22, *p* = 0.24, *I*^2^ = 26%). Furthermore, sensitivity analysis following exclusion of two studies with high risk of bias^15,19^ showed that re-manipulation was in favor of BEC with statistical significance (RR = 0.3, 95% CI = 0.19, 0.48) (Figure 4).

### Cast-related complications

**Figure 5.**
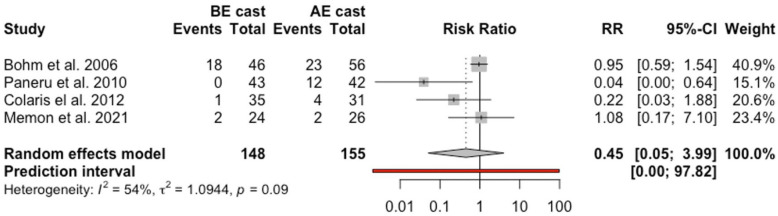
Forest plot: pooled relative risk for cast-related complications for BEC versus AEC treatment.

Five studies reported data regarding cast-related complications with a total of 411 children.^8,14,15,18,24^ Forty out of 202 children (19.8%) had cast-related complications in the BEC group compared to 64 out of 209 children in AEC group (30.6%). Analysis of cast-related complication rate demonstrated that BEC treatment was associated with a pooled RR of 0.59 (95% CI = 0.16, 2.22, *p* = 0.17, *I*^2^ = 38%). This was also not statistically significant. Sensitivity analysis following exclusion of one study with high risk of bias^15^ failed to show a statistical difference between both groups (Figure 5).

Cast complications included reinforcement for cast breakdown, change for loosening or breakdown, cast splitting for swelling, reasons of comfort, and cast falling off. Other complications included excoriation in the elbow crease and transient neuropraxia of the superficial radial nerve.

### Other sensitivity analysis

Further sensitivity analysis with the exclusion of the studies with patients older than 12 years of age^9,14,16^ revealed an overall RR of 0.86 (95% CI = 0.48, 1.54, *p* = 0.05, *I*^2^ = 58%) for loss of fracture reduction in favor of BEC treatment, but this did not reach a statistical significance (*p* = 0.51). The other outcome results and overall RRs altered only minimally.

## Discussion

The most important findings of this meta-analysis were the statistically significant difference of the sensitivity analysis of high-quality studies in the reported outcomes of displaced distal forearm fractures in children who underwent BEC compared to AEC treatment in terms of loss of fracture reduction and re-manipulation. The meta-analysis also suggests a treatment effect favoring BEC with regard to plaster-related complications as well; however, this lacked statistical significance. The relatively small sample sizes and article heterogeneity further confounded the capability to notice a benefit. This heterogeneity can be noticed in the inclusion criteria, the frequency and duration of follow-up, and the reported outcomes including loss of fracture reduction and re-manipulation in the included studies. Other outcomes, such as joint range of motion, functional outcomes, and return to normal activities, were not pooled due to the variability in time points and outcome measures. The rationale behind this meta-analysis was that there is a significant lack of agreement on the indications of cast type application regarding the treatment of displaced distal forearm fractures in pediatric patients.^26–29^ In a survey, Laaksonen et al.^27^ reported possible overtreatment and practice variation for treatment of completely displaced distal radius fractures in pediatric patients. In this survey, the majority of surgeons (84% of 213 participating surgeons) chose an AEC to immobilize distal forearm fractures, although earlier reports showed that AEC does not retain the alignment of displaced distal forearm fractures in children any better than BEC.

All included studies explicitly mentioned that the fractures were displaced. Several questions arise as to how best to define a loss of fracture reduction and the need for re-manipulation and at what specific time point. In our analysis, we took the number of loss of fracture reduction and the need for re-manipulation as specified by the authors. We excluded studies with high risk of bias and children more than 12 years of age in our sensitivity analyses. This was based on analyzing high-quality studies and considering that adolescent fractures might be treated as adult injuries that require surgical treatment to achieve and maintain fracture reduction.

Loss of fracture reduction is the most common complication after closed reduction and cast immobilization in the management of displaced distal forearm fractures in children.^3^ Hafeez et al.^19^ showed in their subgroup analyses adjusting for age group (4–8 or 9–12 years) and involvement of bone (radius only, ulna only, or both bones) significantly lowered the rate of loss of fracture reduction in the BEC group that was evident in the 4–8 years group, 9–12 years group, and both forearm bone group. Webb et al.^9^ suggested that AEC treatment was technically more difficult to apply which resulted in poor molding around the forearm and associated with loss of fracture reduction. The long-term study of Colaris et al.^14^ described no statistically significant differences between both groups at 7-year follow-up.^25^

Re-manipulation was performed with varying reported rates between the studies. Indications for re-manipulation after loss of fracture reduction were not clearly reported in all studies. In the study of Bohm et al.,^8^ only a very small number of children underwent re-manipulation despite meeting the criteria of loss of fracture reduction. The authors suggested that this was due to the assumption that forearm fractures in children have the capability to remodel with growth which will lead to a final satisfactory outcome. Similarly, Webb et al.^9^ reported that none of the patients who lost fracture reduction in both groups underwent re-manipulation and concluded that loss of reduction in this population has minimal clinical relevance due to excellent remodeling potential. Furthermore, Seiler et al.^16^ stated that the confidence in a satisfactory outcome might explain the low rate of re-manipulation in their study due to the high remodeling potential.

## Limitations

The limitations of this meta-analysis include the low number of available RCTs (total of nine studies), the lack of ability to pool all available data and studies with differences in time points and reported outcome measures, the unknown bias in the primary studies, and the inclusion of articles published only in English.

Review of quality demonstrated methodological limitations in most of the included studies, with poor quality of reporting in three studies compared to the others. Studies of low quality have been shown to overestimate the treatment effect compared with high-quality trials.^30^ Hence, the simplest method is to evaluate trials on specific domains of quality that are most relevant to the control of bias for that particular study.

Another limitation was the lack of a standard definition of loss of fracture reduction between studies. Although most of the studies^8,9,14–16,18,24^ had specified criteria for loss of fracture reduction, these criteria were not adjusted for age, except in the studies of Colaris et al.^14^ and Seiler et al.^16^ In addition, we could not specify the exact reason for the decision of re-manipulation of fracture(s) as not all of the fractures that lost reduction were re-manipulated. We contacted the corresponding authors of all the studies to provide us with indications of re-manipulation to avoid underestimating the actual number of fractures that required re-manipulation.

## Conclusion

Our results are compatible with the literature in that any comparative treatment effect is likely to be minor, and reproducing this study designs with higher power may only serve to render a clinically insignificant difference statistically significant. Further attention should be oriented at good methodological-quality articles with adequate follow-up, and priority should be linked to specific patient-centered outcome measures. Finally, our meta-analysis demonstrated that BEC treatment is a safe and effective alternative to AEC treatment of displaced distal forearm fractures in children. It was evident that BEC treatment does not lead to increased loss of fracture reduction and re-manipulation rates and plaster-related complications and should be the mainstay for displaced distal forearm fractures in children.
